# Assistive technology: autonomous wheelchair in obstacle-ridden environment

**DOI:** 10.7717/peerj-cs.725

**Published:** 2021-11-03

**Authors:** Sandeep Ameet Kumar, Jito Vanualailai, Avinesh Prasad

**Affiliations:** School of Information Technology, Engineering, Mathematics, and Physics, The University of the South Pacific, Suva, Fiji

**Keywords:** Wheelchair, Assistive technology, Autonomous system, Collision avoidance, Stabilizing controllers, Motion planning, Lyapunov stability

## Abstract

The benefits for the advancement and enhancement of assistive technology are manifold. However, improving accessibility for persons with disabilities (PWD) to ensure their social and economic inclusion makes up one of the major ones in recent times. This paper presents a set of new nonlinear time-invariant stabilizing controllers for safe navigation of an autonomous nonholonomic rear-wheel drive wheelchair. Autonomous wheelchairs belong to the category of assistive technology, which is most sought in current times due to its usefulness, especially to the less abled (physically and/or cognitively), hence helping create an inclusive society. The wheelchair navigates in an obstacle-ridden environment from its start to final configuration, maintaining a robust obstacle avoidance scheme and observing system restrictions and dynamics. The velocity-based controllers are extracted from a Lyapunov function, the total potentials designed using the Lyapunov based Control Scheme (LbCS) falling under the classical approach of the artificial potential field method. The interplay of the three central pillars of LbCS, which are safety, shortness, and smoothest course for motion planning, results in cost and time effectiveness and the velocity controllers’ efficiency. Using the Direct Method of Lyapunov, the stability of the wheelchair system has been proved. Finally, computer simulations illustrate the effectiveness of the set of new controllers.

## Introduction

Information and Communications Technologies (ICT) has shaped the global transformation into information societies since the late 20th century. ICT is continuing to evolve with the introduction of newer, more enhanced, sophisticated, and targeted technologies. ICT tools have become an indispensable component of everyday life and for every human endeavor. Most sectors, such as socio-economic, political, educational, medical, and healthcare sectors, are integrating innovative ICT tools developed by the industries to their systems and processes to secure solutions tailored to their needs. The inclusion of wireless and sensor technologies have resulted in improved and new designs of assistive technologies. According to the Individuals with Disabilities Education Act (2019), assistive technology device refers to “any item, piece of equipment, or product system, whether acquired commercially off the shelf, modified, or customized, that is used to increase, maintain, or improve functional capabilities of a child with a disability”.

These assistive technologies allow people with severe paralysis to interact and communicate with other industry developed devices such as television set, radio, computer, laptop, and tablets. The objective is to extend the remaining abilities of a person living with severe disabilities to perform limited activities (Moon, Baker & Goughnour, 2019; Assaf et al., 2018; Utkal, Mohammed & Shivneel, 2017). For those having physical debility, mobility is a luxury for them. It is an issue for those who have walking disabilities, such as the elderly, physically disabled, and weak medical care patients. The wheelchair is one of the most frequently used assistive technology in recent and past decades to aid mobility for people with walking disabilities (Borges et al., 2018). Consequentially, a high degree of research work has been in progress to design user-friendly, collision-free electrical and smart wheelchairs such as joystick controlled (Hartman & Nandikolla, 2019), smartphone controlled (Borges et al., 2018; Gulpanich, Petchhan & Wongvanich, 2018), gesture controlled (eye movement (Solea et al., 2015; Bharath, Sreenidhi & Tarun, 2018; Eid, Giakoumidis & Saddik, 2016; Maule et al., 2016), oral motion (Saitoh, Takahashi & Konishi, 2007), and head motion (Tomari, Kobayashi & Kuno, 2014)), voice controlled (Chen et al., 2008), brain controlled (Xin et al., 2018; Ng & Goh, 2020), blue-tooth controlled (Qin, Li & Nawaz, 2018), multiple control interfaces (Yashoda et al., 2018; Shahnaz et al., 2017), and semi-autonomous controlled (Tomari, Kobayashi & Kuno, 2012) to improve mobility, which can enhance the quality of life for the elderly and the disabled (Takahashi & Murakami, 2018; Bumuller & Skerl, 2018; Chen et al., 2008). The design of wheelchairs has to be such that the safety and comfortability of its users should not be compromised, rather enhanced (Tomari, Kobayashi & Kuno, 2014). It should also uplift the lifestyle of its users in terms of social interactions, access to education and opportunities, and the ability to exercise their knowledge and skills (Shahnaz et al., 2017). While wheelchairs have been most sought for mobility, integration of ICTs and controller laws are gradually transforming these assistive vehicles into smart, intelligent or autonomous robots. Futuristic models seek to be independent of controls that require human interaction, and transform them into fully automated systems. For instance, those suffering from severe motor impairment face difficulties in operating electric or smart wheelchairs that require user input or interaction (Assaf et al., 2018; Tomari, Kobayashi & Kuno, 2012) will prefer automated wheelchairs instead. Electronic and smart wheelchairs (Assaf et al., 2018; Borges et al., 2018; Hartman & Nandikolla, 2019; Gulpanich, Petchhan & Wongvanich, 2018; Solea et al., 2015; Bharath, Sreenidhi & Tarun, 2018; Eid, Giakoumidis & Saddik, 2016; Maule et al., 2016; Saitoh, Takahashi & Konishi, 2007; Tomari, Kobayashi & Kuno, 2014; Chen et al., 2008; Xin et al., 2018; Ng & Goh, 2020; Qin, Li & Nawaz, 2018; Yashoda et al., 2018; Shahnaz et al., 2017; Tomari, Kobayashi & Kuno, 2012; Takahashi & Murakami, 2018; Bumuller & Skerl, 2018; Chen et al., 2008) present in the literature are mostly experimental types, and the stability of these systems are missing together with robust control laws which can make these robots fully autonomous. Recently, Andaluz et al. (2015) and Herrera et al. (2018) presented the kinematic and dynamic modeling of a human-wheelchair system which is capable of performing positioning and path-following tasks in a shared environment. Moreover, using the path following control laws, the notion of graceful motion, that is, the movement that is safe, smooth, fast, and intuitive, for a robotic wheelchair presented in Gulati & Kuipers (2008) concentrated on a specific task of narrow way clearance. An assistive collision avoidance method developed in Gillham & Howells (2015) based on potential fields required human interaction for a powered wheelchair that allows the user to navigate safely.

In this paper, a set of new nonlinear time invariant stabilizing velocity-based controllers is developed which facilitates navigation of an autonomous wheelchair robot in an obstacle-ridden environment, maintaining a robust obstacle avoidance scheme and observing system restrictions and dynamics. Stabilizing controllers are derived from a total potential function developed using the Lyapunov based Control Scheme (LbCS) which has been deployed successfully in literature to find feasible and stabilizing solutions for a wide spectrum of applications (Sharma, Vanualailai & Singh, 2012; Sharma, Vanualailai & Singh, 2014; Kumar, Vanualailai & Sharma, 2015a; Kumar et al., 2016; Devi et al., 2017; Sharma, Vanualailai & Singh, 2015; Sharma, Raj & Vanualailai, 2018; Sharma, Vanualailai & Prasad, 2017; Sharma et al., 2018; Raghuwaiya, Sharma & Vanualailai, 2018; Sharma, Vanualailai & Prasad, 2011; Kumar, Vanualailai & Sharma, 2015b; Kumar & Vanualailai, 2017; Kumar et al., 2021b; Prasad et al., 2020). Interaction of the three main pillars of LbCS, which are safety, shortness, and smoothest path for motion planning, bring about cost and time effectiveness and efficiency of the velocity controllers. From the authors’ viewpoint, this is the first instance the methodology is applied to wheelchairs.

The major contributions of this paper are:

1. Design of a set of new stabilizing nonlinear time-invariant continuous controllers for an autonomous wheelchair robot for navigation observing system restrictions and limitations through the use of LbCS. From the authors’ point of view, this is the first time such stabilizing continuous velocity-based controllers are derived for autonomous wheelchairs in the sense of Lyapunov. Similar obstacle avoidance scheme was presented in Rösmann, Hoffmann & Bertram (2017), however, using inequalities, whereas in Darweesh et al. (2017), planning and tracking are split, and the mechanical singularities and constraints are not considered within the path planner. Similarly, Lazarowska (2019) does not consider the mechanical singularities and constraints despite of effective solutions in terms of the path length and run time of the algorithm.
2. Obstacle avoidance in an environment without any interaction between the user and the wheelchair. In contrast, the wheelchairs reported in the literature mostly require human interaction for it to be able to reach the rider’s destination safely. Wheelchairs that require human interaction could collide with obstacles as weak motor patients sometimes would not be able to react on time. For instance, while controlling button-based, voice-based (Gulpanich, Petchhan & Wongvanich, 2018; Chen et al., 2008; Qin, Li & Nawaz, 2018; Yashoda et al., 2018; Shahnaz et al., 2017), head movement-based (Borges et al., 2018; Tomari, Kobayashi & Kuno, 2014), oral motion controlled (Saitoh, Takahashi & Konishi, 2007), joystick-based (Borges et al., 2018; Yashoda et al., 2018), eyeball sensed (Borges et al., 2018; Solea et al., 2015; Eid, Giakoumidis & Saddik, 2016; Maule et al., 2016) and EEG signaled (Xin et al., 2018; Ng & Goh, 2020) wheelchairs. Moreover, the wheelchairs, such as the ones reported in references (Borges et al., 2018; Gulpanich, Petchhan & Wongvanich, 2018; Solea et al., 2015; Eid, Giakoumidis & Saddik, 2016; Maule et al., 2016; Saitoh, Takahashi & Konishi, 2007; Tomari, Kobayashi & Kuno, 2014; Chen et al., 2008; Xin et al., 2018; Ng & Goh, 2020; Qin, Li & Nawaz, 2018; Yashoda et al., 2018; Shahnaz et al., 2017; Tomari, Kobayashi & Kuno, 2012), that require human interactions cannot perform the simplest of the tasks of moving from one place to the other for persons with special disabilities who are incapable of any interactions or cannot provide any commands to the wheelchairs.
3. Optimal route amidst obstacles, constraints and restrictions from start to target points inherently guaranteed by the LbCS.

The remainder of the paper is organized as follows: “Lyapunov-Based Control Scheme” gives a brief description of the LbCS. In “Kinematic Model of a Nonholonomic Wheelchair”, kinematic equations of a rear two wheels driven wheelchair robot with two front castor wheels based on a geometric model are developed assuming there is no lateral slip motion on the wheels, and there is pure rolling. “Velocity Controllers of the Wheelchair Robot”, the nonlinear time-invariant stabilizing velocity controllers are derived for the wheelchair robot using LbCS. In “Angular Velocities and their Limitations”, the restrictions on the angular velocities of the wheels of the wheelchair robot are discussed. The stability analysis of the wheelchair robot system is presented in “Stability Analysis”. The simulation studies are presented in “Simulation Results”. Finally, a discussion on the results presented is provided in “Discussion” and the research is concluded with a brief on future undertakings in “Conclusion”.

## Lyapunov-based control scheme

This research utilizes an artificial potential field technique known as the Lyapunov-based control scheme. The development of attractive and repulsive potential field functions is the primary intention of LbCS. Subsequently, these functions are part of a total potential function called the Lyapunov function from which one could extract the time-invariant nonlinear velocity or acceleration controllers (Sharma, Vanualailai & Singh, 2014; Sharma, Vanualailai & Singh, 2015; Sharma, Raj & Vanualailai, 2018; Sharma, Vanualailai & Prasad, 2017; Sharma et al., 2018; Kumar et al., 2021b; Prasad et al., 2020; Chand et al., 2020; Prasad et al., 2021; Kumar et al., 2021a). Using LbCS, designing controllers is easy, and the controllers are continuous, which are its strength. It is easy to include control conditions, specifications, inequalities, and mechanical constraints of mechanical systems in the controllers through developing mathematical functions when applying LbCS. The main disadvantage of LbCS is that algorithm singularities (local minima) can be introduced. In practical applications, continuity has to be discretized, and only then asymptotic stability could be shown. The reader is referred to Sharma, Vanualailai & Singh (2014) and Sharma, Vanualailai & Prasad (2017) for a detailed account of the LbCS.

**Figure 1:**
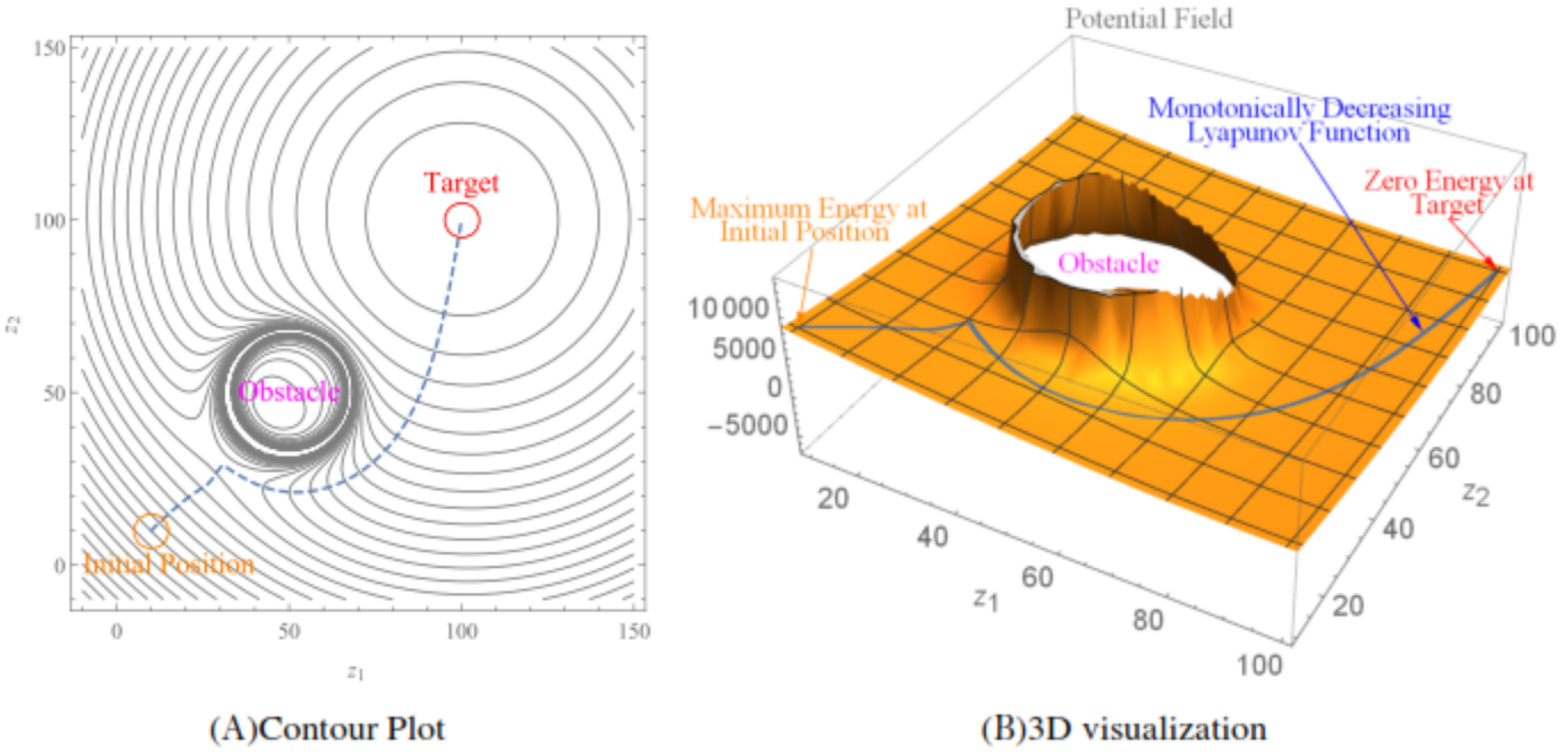
An illustration of the Lyapunov-based control scheme.

An illustration of the LbCS is given utilizing Figs. 1A and 1B. Figure 1A shows the contour plot generated over a workspace:amp:minus; −10 < *z*_2_ < 150 and −10 < *z*_1_ < 150 for a robot whose initial position is at (10, 10). The dashed line is the robot’s trajectory from its initial position to its target position (100, 100), which shows the robot avoids the obstacle positioned at (50, 50) with radius 10. Figure 1B shows the 3D visualization of the attractive and repulsive potential fields. The blue line shows the Lyapunov function, which shows that the energy of the robot is monotonically decreasing and is zero at the target position.

## Kinematic model of a nonholonomic wheelchair

**Definition 3.1** A rear two wheels driven wheelchair with two front castor wheels is a disk with radius *r*_*w*_ and is positioned at center (*x, y*). A wheelchair is precisely described as the set

(1) $${V} = \{ ({z_1},{z_2}) \in {{\rm {\mathbb {R}}}^2}:({z_1} - x{)^2} + {({z_2} - y)^2} \le r_w^2\} .$$

**Figure 2:**
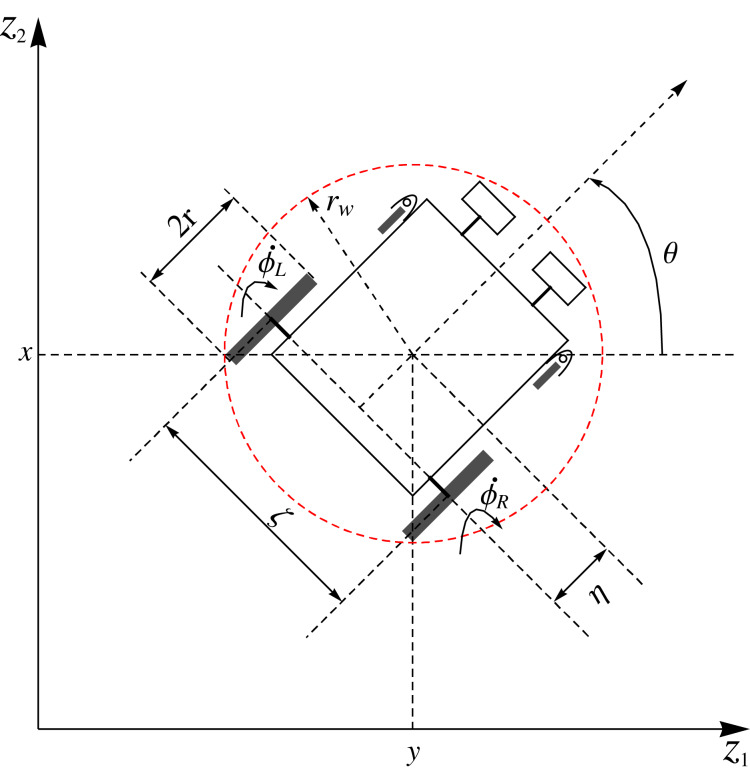
Geometric model of a two rear wheels driven wheelchair with two front castor wheels at an orientational angle *θ*.

A two rear wheels driven wheelchair robot with two front castor wheels is shown in Fig. 2. The two rear drive wheels of radius *r* are on opposite ends of a wheelbase of length *ς*. The angle *θ* is the orientational angle of the wheelchair robot with respect to *z*_1_-axis of the *z*_1_*z*_2_ cartesian plane. The centre of the wheelchair robot is at (*x*, *y*) which is at a distance of *η* with orientational angle *θ* from the centre of the two rear diametrically opposed wheels. The angular velocities of the rear right and left wheels are ${\dot \phi _R} = {\upsilon _R}$ and ${\dot \phi _L} = {\upsilon _L}$, respectively. To ensure that the wheelchair robot steers safely pass obstacles (either moving or static obstacles), the wheelchair is enclosed by a smallest possible circle. As shown in Fig. 2, the wheelchair robot is enclosed by a protective circular region centered at (*x*, *y*), with radius ${r_w}: = \sqrt {{{(\textstyle{\zeta \over 2})}^2} + {{(\eta + r)}^2}}$. Hence, the configuration vector for the wheelchair robot is,

(2) $${\bf q} = [x,y,\theta ,{\phi _R},{\phi _L}].$$

It has been assumed that there are no uncertainties in the kinematic parameters of the wheel radius and the wheelbase. Moreover, assuming that there is pure rolling and no lateral slip motion on the two rear wheels, with respect to (*x*, *y*) the following constraints are obtained:

(3) $$\dot y\cos \theta - \dot x\sin \theta - \dot \theta \eta = 0$$

(4) $$\dot x\cos \theta + \dot y\sin \theta + \displaystyle{\zeta \over 2}\dot \theta - r{\dot \phi _R} = 0$$

(5) $$\dot x\cos \theta + \dot y\sin \theta - \displaystyle{\zeta \over 2}\dot \theta - r{\dot \phi _L} = 0.$$

This is in line with the derivations obtained in the literature by Solea et al. (2015) and Dhaouadi & Hatab (2013). These are the non-holonomic constraints of the wheelchair robot which needs to be appropriately factored into the kinematic model of the robot. The kinematic model, of the robot with respect to its center $(x,y) \in {{\rm {\mathbb {R}}}^2}$ is derived as

(6) $$\left. {\hskip-1.7pc}{\matrix{ {} \hfill {} \hfill {\dot x = \displaystyle{r \over \zeta }({\upsilon _R}(\displaystyle{\zeta \over 2}\cos \theta - \eta \sin \theta ) + {\upsilon _L}(\displaystyle{\zeta \over 2}\cos \theta + \eta \sin \theta )),} \hfill {} \hfill \cr {} \hfill {} \hfill {\dot y = \displaystyle{r \over \zeta }({\upsilon _R}(\displaystyle{\zeta \over 2}\sin \theta + \eta \cos \theta ) + {\upsilon _L}(\displaystyle{\zeta \over 2}\sin \theta - \eta \cos \theta )),} \hfill {} \hfill \cr {} \hfill {} \hfill {\dot \theta = \displaystyle{r \over \zeta }({\upsilon _R} - {\upsilon _L}),} \hfill {} \hfill \cr {} \hfill {} \hfill {{{\dot \phi }_R} = {\upsilon _R},} \hfill {} \hfill \cr {} \hfill {} \hfill {{{\dot \phi }_L} = {\upsilon _L}.} \hfill {} \hfill \cr } } \right\}$$

In a two-dimensional space, the position of a wheelchair robot can be described by its translational components. Let the position of a wheelchair at time *t* $\geq$ 0 be **x** = (*x*(*t*), *y*(*t*)) and orientational angle *θ = θ*(*t*), with (*x*(*t*_0_), *y*(*t*_0_)) =: (*x*_0_, *y*_0_) and *θ*(*t*_0_) *= θ*_0_ as initial conditions. At *t* ≥ 0, let (ρ(*t*), ω(*t*)) := (*x′*(*t*), *y′*(*t*)) be the instantaneous velocity of the wheelchair robot. We have thus a system of first-order ODEs for the wheelchair robot:

(7) $$x ^{\prime}(t) = \rho(t),\ y^{\prime}(t) = \omega(t),$$

assuming the initial conditions at $t = {t_0} \ge 0$ as ${x_0}: = x({t_0}),\;{y_0}: = y({t_0})).$ Suppressing $t,$ we let ${\bf x}: = (x,y) \in {{\rm {\mathbb {R}}}^2}$ and let ${{\bf x}_0}: = {\bf x}({t_0}): = ({x_0},{y_0}) \in {{\rm {\mathbb {R}}}^2}$. If the instantaneous velocity $(\rho ,\omega )$ has a state feedback law of the form

$\rho (t): = - \mu f({\bf x}(t)),$

$\omega (t): = - \varphi g({\bf x}(t)),$

for some scalars $\mu ,\varphi > 0$ and some functions $f({\bf x}(t))$ and $g({\bf x}(t))$ to be constructed appropriately later, and if we define ${\bf G}({\bf x}): = ( - \mu f({\bf x}), - \varphi g({\bf x})) \in {{\rm {\mathbb {R}}}^2}$, then the wheelchair robot is represented by

(8) $${\bf \dot x} = {\bf G(x)},\;\;{\bf x}({t_0}) = {{\bf x}_0}.$$

The equilibrium point for the wheelchair robot is ${{\bf x}_e} = ({x_e},{y_e}) \in {{\rm {\mathbb {R}}}^2}$.

## Velocity controllers of the wheelchair robot

Consider *a priori* known workspace cluttered with $q \in {\rm {\mathbb {N}}}$ stationary obstacles. The wheelchair robot governed by system (8) has to maneuver to its target, avoiding collision with static obstacles.

**Definition 4.1** The ${k^{th}}$ solid stationary obstacle is a disk with center ${{\bi{x}}_{{O_k}}} = ({o_{k1}},{o_{k2}})$ and radius ${r_{{O_k}}} > 0$. It is described as the set

(9) $${O_k}: = \{ ({z_1},{z_2}) \in {{\rm {\mathbb {R}}}^2}:({z_1} - {o_{k1}}{)^2} + {({z_2} - {o_{k2}})^2} \le r_{{O_k}}^2\} .$$

**Definition 4.2** The target for the wheelchair robot is ${{\bi{x}}_\tau }$. It is a disk with center ${{\bi{x}}_\tau } = (a,b)$ and radius ${r_w}$. It is described as the set

(10) $$\tau : = \{ ({z_1},{z_2}) \in {{\rm {\mathbb {R}}}^2}:({z_1} - a{)^2} + {({z_2} - b)^2} \le r_w^2\} .$$

### Components of the Lyapunov function

In the Lyapunov function to be proposed for the total potential, the following attractive and repulsive potential functions will be included.

#### Target attraction

**Figure 3:**
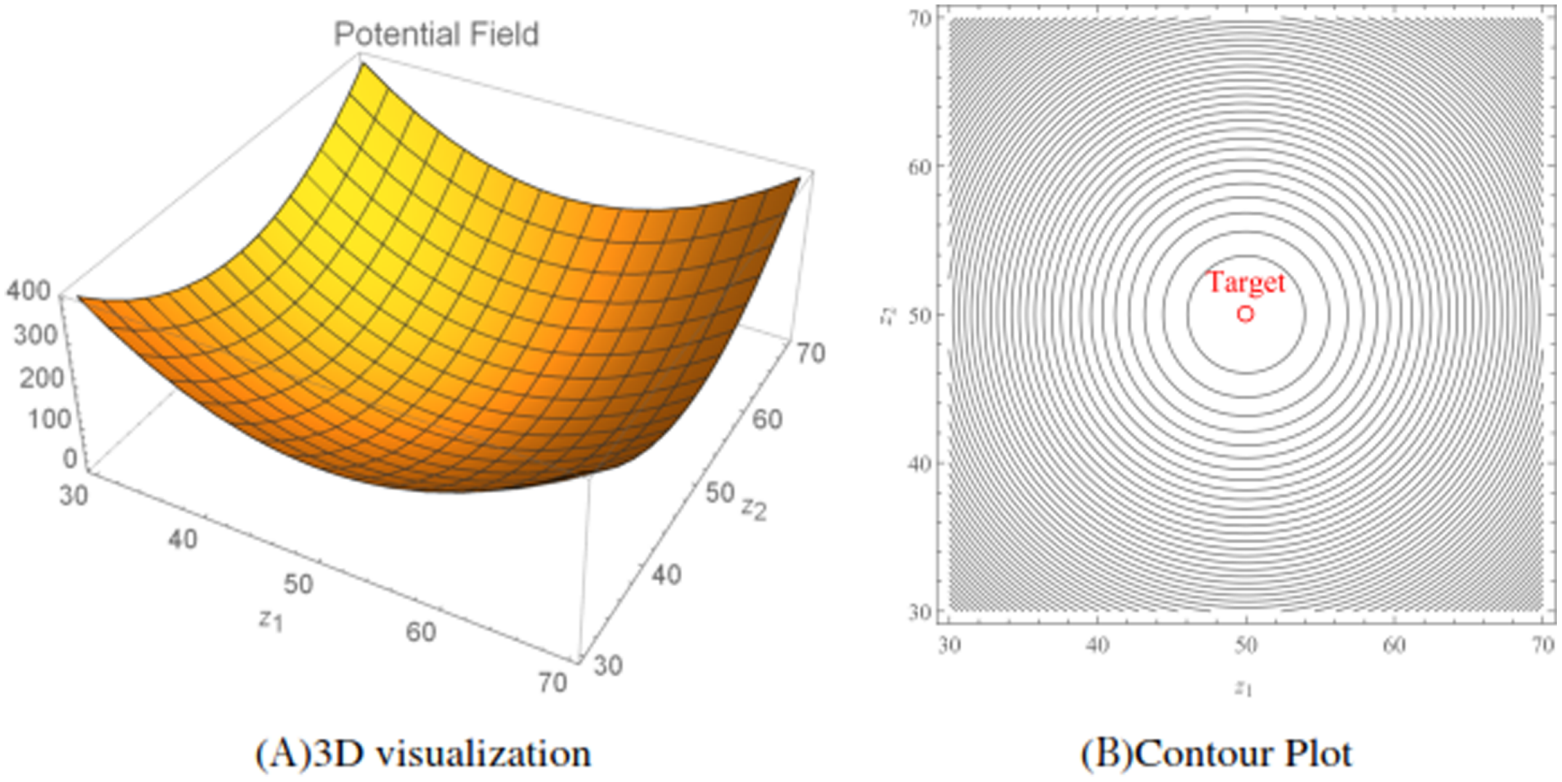
The attractive potential fields and the corresponding contour plot generated using the attractive potential function (11). The position of the target is located at (50, 50) with a *r_w_* = 0.5. The target convergence parameter *α* = 1.

To ensure that the wheelchair robot converges to its equilibrium position, we shall utilize the target attraction potential function

(11) $${U_{att}}({\bf x}): = \displaystyle{1 \over 2}\alpha {{\bf e}_1}^2,$$

where $\alpha > 0$ is the target convergence parameter, and ${{\bf e}_1} = \| {{\bf x} - {x_\tau }}\|$ is the distance between the wheelchair robot position and the target at any arbitrary time. The target convergence parameter, $\alpha,$ can be considered as a measurement of the strength of attraction between the wheelchair robot, $\bi x$, and its target, ${{\bi{x}}_\tau }$. A small value of the parameter indicates a slower convergence of the wheelchair robot to its target. An illustration of the total potentials for the function (11) is shown in Fig. 3A, while Fig. 3B shows the corresponding contour plot generated over a workspace $30 < {z_1} < 70$ and $30 < {z_2} < 70$.

#### Stationary obstacle avoidance

**Figure 4:**
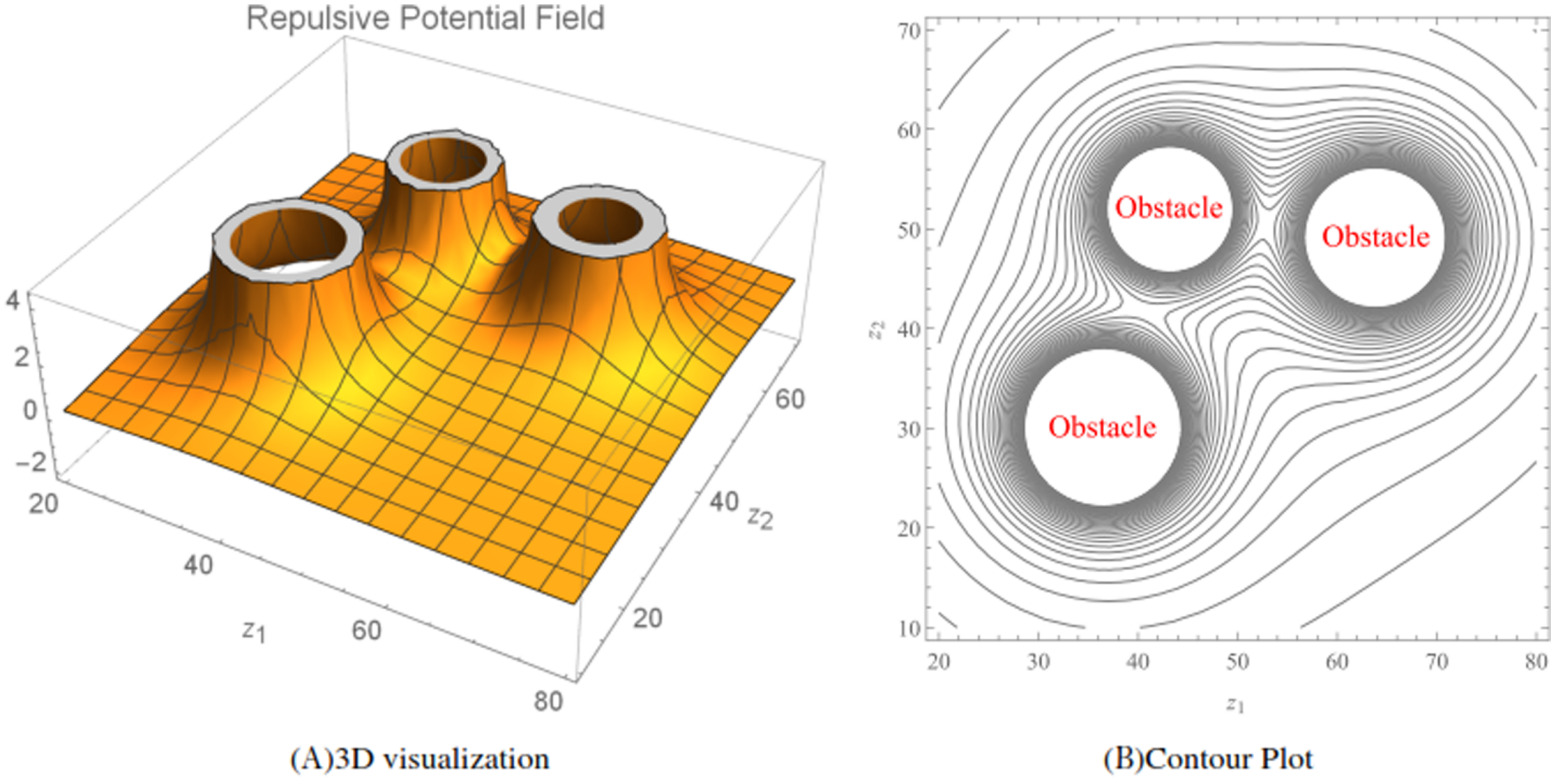
The repulsive potential fields and the corresponding contour plot generated using the repulsive potential function (13). The radius of the obstacles were randomized between three and five and *r_w_* = 1. The obstacle avoidance parameter *β_k_* for *k* = 1, 2 and 3 were randomized between 30 and 70.

For the purpose of avoiding possible collisions with the ${k^{th}}$ stationary solid obstacle governed by Eq. (9), the following obstacle avoidance function will be utilized for the wheelchair robot:

(12) $${W_k}({\bf x}) = \displaystyle{1 \over 2}\left[ {{{\bf e}_2}^2 - {{\left( {{r_{{O_k}}} + {r_w}} \right)}^2}} \right],\quad k \in \{ 1,2,...,q\} .$$

where ${{\bf e}_2} = \left\| {{\bf x} - {{\bf x}_{{O_k}}}} \right\|$ is the distance between the wheelchair robot position and the centre of the obstacle at any arbitrary time. Thus, the total repulsive potential is given by

(13) $${U_{rep}}({\bf x}) = \sum_{k = 1}^{q}\displaystyle{{{\beta _k}} \over {{W_k}({\bf x})}}$$

where ${\beta _k} > 0$ is the obstacle avoidance parameter. At large distances between the wheelchair robot and the ${k^{th}}$ obstacles, ratio (13) is negligible. Now, consider the situation where the wheelchair robot approaches the ${k^{th}}$ obstacle. In this case, ${W_k}({\bf x})$ decreases, and the ratio (13) increases, with ${\beta _k} > 0$ acting as a *obstacle avoidance parameters*, that is a measurement of the strength of interaction between the wheelchair robot and the ${k^{th}}$ obstacle. Here, the ratio (13) acts as an *collision-avoidance function* because it can be allowed to increase in value (corresponding to avoidance) as the wheelchair robot approaches the stationary obstacle. An illustration of the total repulsive potentials for three randomly generated obstacles ( $q = 3$) for the function (13) is shown in Fig. 4A, while Fig. 4B shows the corresponding contour plot generated over a workspace $20 < {z_1} < 80$ and $10 < {z_2} < 70$.

#### Auxiliary function

To ensure that the wheelchair robot converges to its target and guarantee that the nonlinear velocity controllers vanish at the target consider the auxiliary function of the form

(14) $$H({\bf x}): = \displaystyle{1 \over 2}{{\bf e}_1}^2$$

This auxiliary function will be multiplied to the total repulsive potential.

### A Lyapunov function

Using the attractive and repulsive potentials with the auxiliary function, a total potential called the Lyapunov function, is formed as,

(15) $$L({\bf x}) = {U_{att}}({\bf x}) + H({\bf x}){U_{rep}}({\bf x}).$$

**Figure 5:**
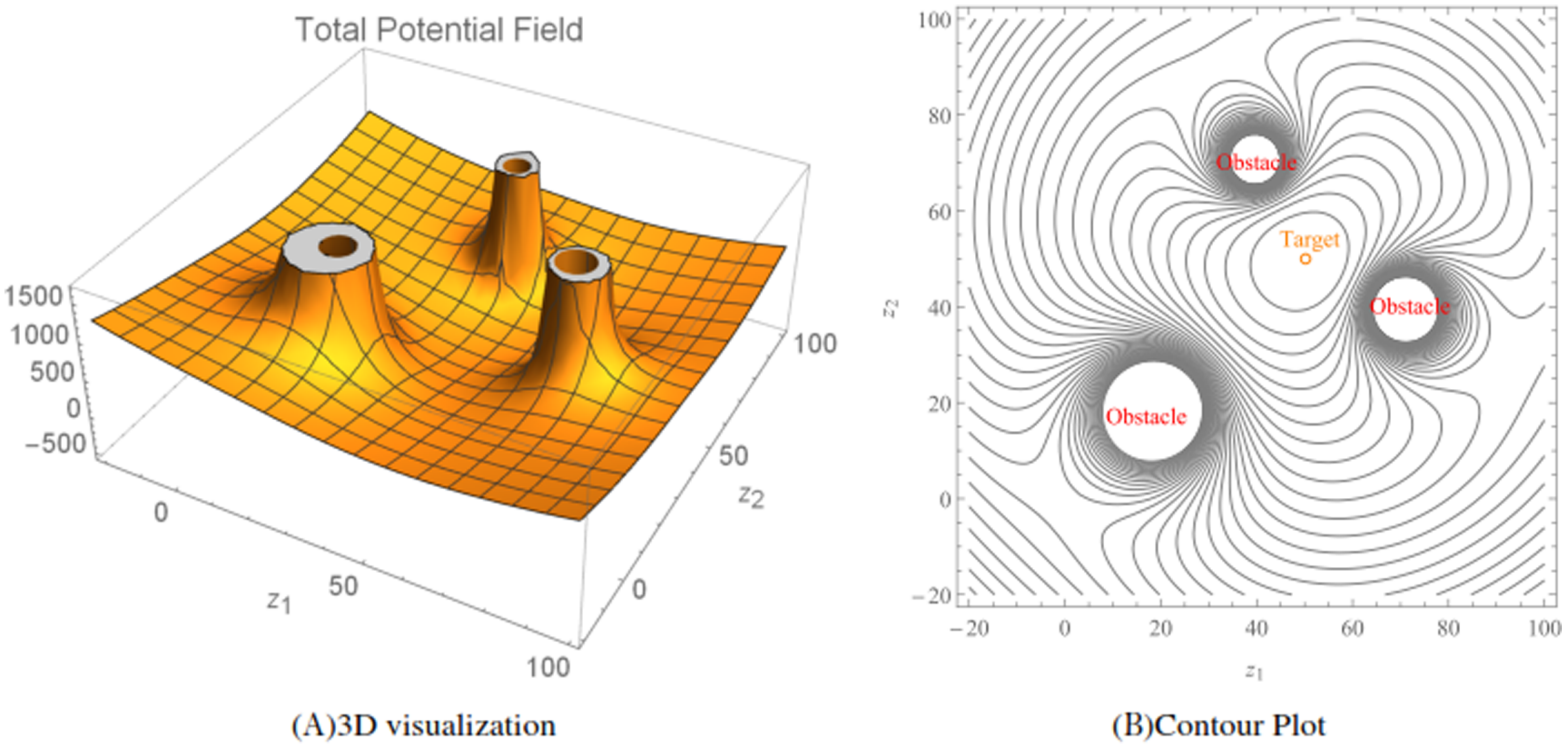
The total potential fields and the corresponding contour plot generated using the Lyapunov function (15). The radius of the obstacles were randomized between two and four, *r_w_* = 1, *α* = 0.2, *β_k_* for *k* = 1, 2 and 3 were randomized between 50 and 70.

An illustration of the total potentials for the Lyapunov function (15) for three randomly generated obstacles and a target situated at (50, 50) is shown in Fig. 5A, while Fig. 5B shows the corresponding contour plot generated over a workspace $- 20 < {z_1} < 100$ and $- 20 < {z_2} < 100$.

### Velocity controllers

Along a trajectory of system (8),

(16) $$\eqalign{\dot L({\bf x}) = \nabla L({\bf x}) \cr= f({\bf x})\dot x + g({\bf x})\dot y, }$$

where

(17) $$f({\bf x}) = \left( {x - a} \right) \left(\alpha + \sum_{k = 1}^{q}\displaystyle{{{\beta _k}} \over {{W_k}({\bf x})}}\right) - \sum\limits_{k = 1}^q {\beta _k}\displaystyle{{H({\bf x})} \over {W_k^2({\bf x})}}\left( {x - {o_{k1}}} \right)$$

and

(18) $$g({\bf x}) = \left( {y - b} \right)\left(\alpha + \sum_{k = 1}^{q}\displaystyle{{{\beta _k}} \over {{W_k}({\bf x})}}\right) - \sum\limits_{k = 1}^q {\beta _k}\displaystyle{{H({\bf x})} \over {W_k^2({\bf x})}}\left( {y - {o_{k2}}} \right).$$

Let there be scalars $\mu > 0$ and $\varphi > 0$. Then the velocity controllers of system (8) are

(19) $$\rho = - \mu f({\bf x})\;{\rm and}\;\omega = - \varphi g({\bf x}).$$

## Angular velocities and their limitations

The system of ODEs (6) is substituted into the time derivative of (15) as shown below:

$\eqalign {\dot L({\bf x}) = f({\bf x}) \cdot \dot x + g({\bf x}) \cdot \dot y \cr= \displaystyle{{rf({\bf x})} \over \zeta }\left({\upsilon _R}\left(\displaystyle{\zeta \over 2}\cos \theta - \eta \sin \theta \right) + {\upsilon _L}\left(\displaystyle{\zeta \over 2}\cos \theta + \eta \sin \theta \right)\right) \cr\quad+ \displaystyle{{rg ({\bf x})} \over \zeta } \left({\upsilon _R}\left(\displaystyle{\zeta \over 2}\sin \theta + \eta \cos \theta \right) + {\upsilon _L}\left(\displaystyle{\zeta \over 2}\sin \theta - \eta \cos \theta \right)\right) \cr= \displaystyle{r \over \zeta }\left(f({\bf x})\left(\displaystyle{\zeta \over 2}\cos \theta - \eta \sin \theta \right) + g\left({\bf x}\right)\left(\displaystyle{\zeta \over 2}\sin \theta + \eta \cos \theta \right)\right){\upsilon _R} \cr\quad+ \displaystyle{r \over \zeta }\left(f({\bf x})\left(\displaystyle{\zeta \over 2}\cos \theta + \eta \sin \theta \right) + g\left({\bf x}\right)\left(\displaystyle{\zeta \over 2}\sin \theta - \eta \cos \theta \right)\right){\upsilon _L}.}$

**Figure 6:**
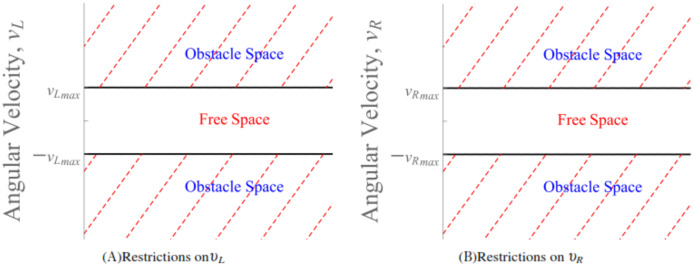
The obstacle space forms the artificial constraints that restricts the angular velocities ${\upsilon _L}$ and ${\upsilon _R}$.

Subsequently, angular velocities of the rear right and left wheels could be defined as

(20) $$\left. {\matrix{ {{\upsilon _R}: = - \displaystyle{{{\kappa _1}r} \over \zeta }\left(f({\bf x})\left(\displaystyle{\zeta \over 2}\cos \theta - \eta \sin \theta \right) + g({\bf x})\left(\displaystyle{\zeta \over 2}\sin \theta + \eta \cos \theta \right)\right),} {} \cr {{\upsilon _L}: = - \displaystyle{{{\kappa _2}r} \over \zeta }\left(f({\bf x})\left(\displaystyle{\zeta \over 2}\cos \theta + \eta \sin \theta \right) + g({\bf x})\left(\displaystyle{\zeta \over 2}\sin \theta - \eta \cos \theta \right)\right),} {} \cr } } \right\}$$

where ${\kappa _1}$ and ${\kappa _2}$ are desired to be some arbitrary continuous positive function of $x$ and $y$, and $f({\bf x})$ and $g({\bf x})$ are defined in (17) and (18), respectively. Moreover, the angular velocities of the rear right and left wheels do have restrictions practically. An illustration of this restriction is shown in Fig. 6. To add on, maximum angular velocities of the left and right wheels of the wheelchair can also be treated as artificial constraints which could be part of the total potential as repulsive potentials. However, the equivalent is to bound the angular velocities. The later is discussed in this paper.

The functions ${\kappa _1} = {\kappa _1}(x,y) > 0$ and ${\kappa _2} = {\kappa _2}(x,y) > 0$ lay an important role in restricting the sizes of ${\upsilon _R}$ and ${\upsilon _L}$, respectively. Given $\chi > 0$ from (20),

(21) $$\left. {\matrix{ {\left| {{\upsilon _R}} \right| \le {\kappa _1}\left(\displaystyle{r \over 2} + \displaystyle{{r\eta } \over \zeta }\right)\left( {\chi + \left| {f({\bf x})} \right| + \left| {g({\bf x})} \right|} \right),} {} \cr {\left| {{\upsilon _L}} \right| \le {\kappa _2}\left(\displaystyle{r \over 2} + \displaystyle{{r\eta } \over \zeta }\right)\left( {\chi + \left| {f({\bf x})} \right| + \left| {g({\bf x})} \right|} \right).} {} \cr } } \right\}$$

If we let ${\upsilon _R}_{max}: = \max \left| {{\upsilon _R}} \right|$ and ${\upsilon _L}_{max}: = \max \left| {{\upsilon _L}} \right|$ be the maximum angular velocities then from (21)

(22) $${\kappa _1}: = \displaystyle{{{\upsilon _R}_{max}} \over {\left(\displaystyle{r \over 2} + \displaystyle{{r\eta } \over \zeta }\right)(\chi + \left| {f({\bf x})} \right| + \left| {g({\bf x})} \right|)}}$$

and

(23) $${\kappa _2}: = \displaystyle{{{\upsilon _L}_{max}} \over {\left(\displaystyle{r \over 2} + \displaystyle{{r\eta } \over \zeta }\right)(\chi + \left| {f({\bf x})} \right| + \left| {g({\bf x})} \right|)}}.$$

## Stability analysis

It is evident that $L({\bf x})$, is positive over the domain

$D(L({\bf x})): = \{ {\bf x} \in {{\rm {\mathbb {R}}}^{2n}}:{W_k}({\bf x}) > 0,\;\forall \,k = \{ 1,2,3, \ldots ,q\} \}$

and with respect to system (6) and the angular velocities of the rear right and left wheels mentioned in (20),

$\dot L({\bf x}) = - \left( {\displaystyle{{\upsilon _R^2} \over {{\kappa _1}}} + \displaystyle{{\upsilon _L^2} \over {{\kappa _2}}}} \right) \le 0.$

$\forall {\bf x} \in D(L({\bf x}))$. At the target, where $(x,y) = (a,b)$, the angular velocities, ${\upsilon _R}$ and ${\upsilon _L},$ are zero because $f({\bf x}) = 0$ and $g({\bf x}) = 0$. It is easy to see that $L({{\bf x}_e}) = 0,L({\bf x}) \gt 0\ \forall\ {\bf x} \ne {{\bf x}_e}$ and $\dot L({\bf x}) \le 0$. Therefore, system (6) is stable.

## Simulation results

Simulations were generated using the Wolfram Mathematica 11.2 software. To achieve the desired results a number of sequential Mathematica commands were executed. System (6) was numerically simulated using RK4 method (Runge–Kutta Method). Due to the inherent nature of the artificial potential field method, which includes LbCS, there is a possibility that some initial conditions can produce trajectories that get trapped in local minima. Firstly, the initial position of the robot relative to the obstacle. For instance, if the robot is near the obstacle then there is a possibility that the robot will move into local minima and get trapped there. Secondly, there is a need to avoid the collinear situation of the robot’s initial position, obstacle position, and target location (Vanualailai, Sharma & Nakagiri, 2008). Such initial conditions are avoided when assigning values to parameters through brute-force.

**Figure 7:**
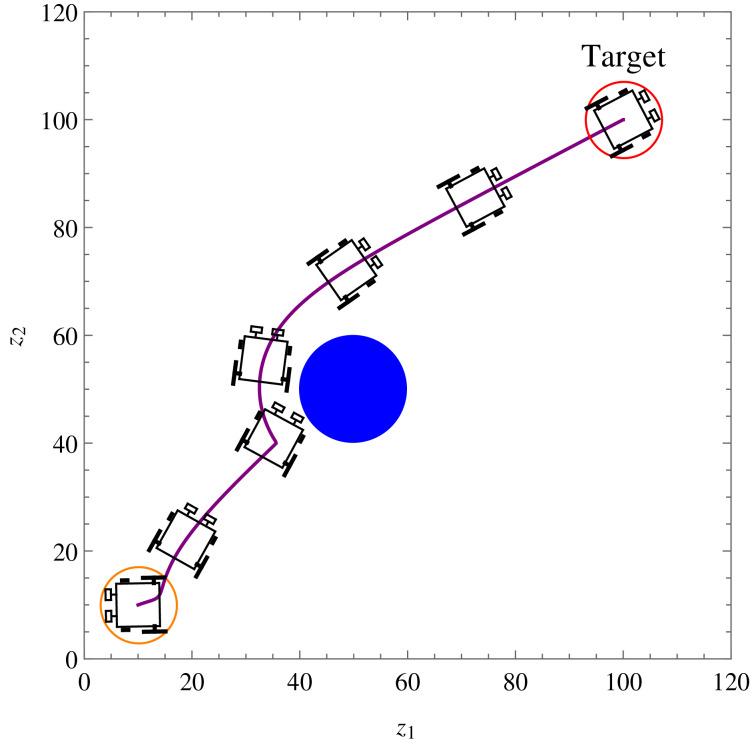
*Example 1*. Position and orientation of the wheelchair robot at *t* = 0, 27, 39, 78, 95, 115 and 140 respectively. The trajectory of (x, y) is shown in solid line.

**Figure 8:**
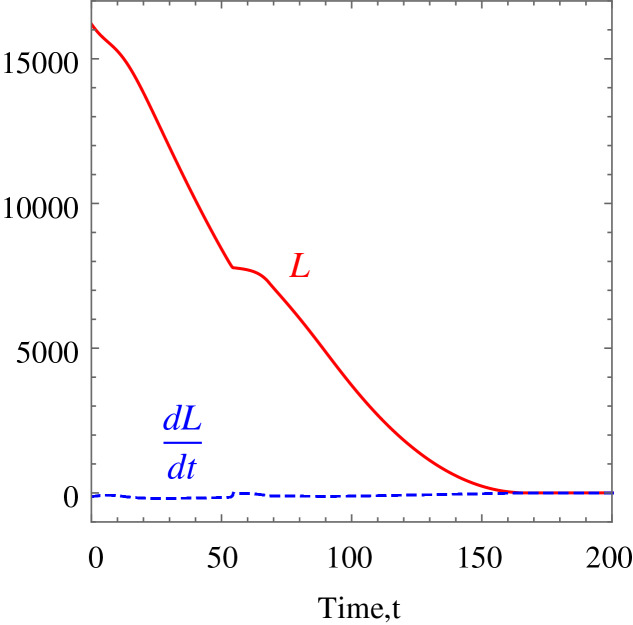
*Example 1*. Monotonically decreasing Lyapunov function and its time derivative.

**Figure 9:**
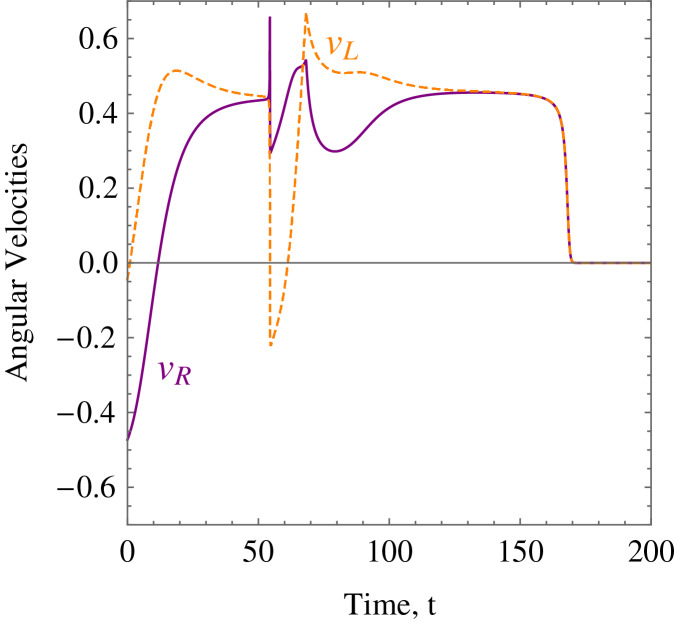
*Example 1*. The angular velocities of the wheelchair robot showing rapid deceleration as it approaches the target.

**Figure 10:**
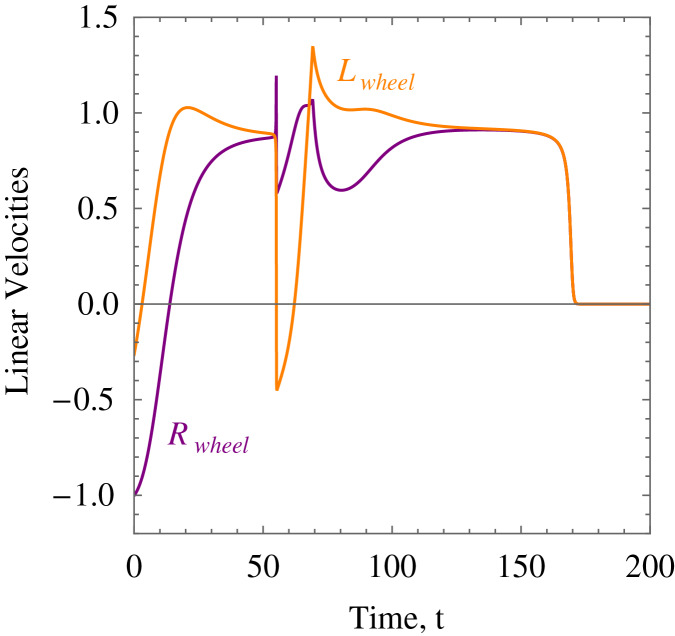
*Example 1*. The linear velocities of the wheels of the wheelchair robot showing rapid deceleration as it approaches the target.

**Figure 11:**
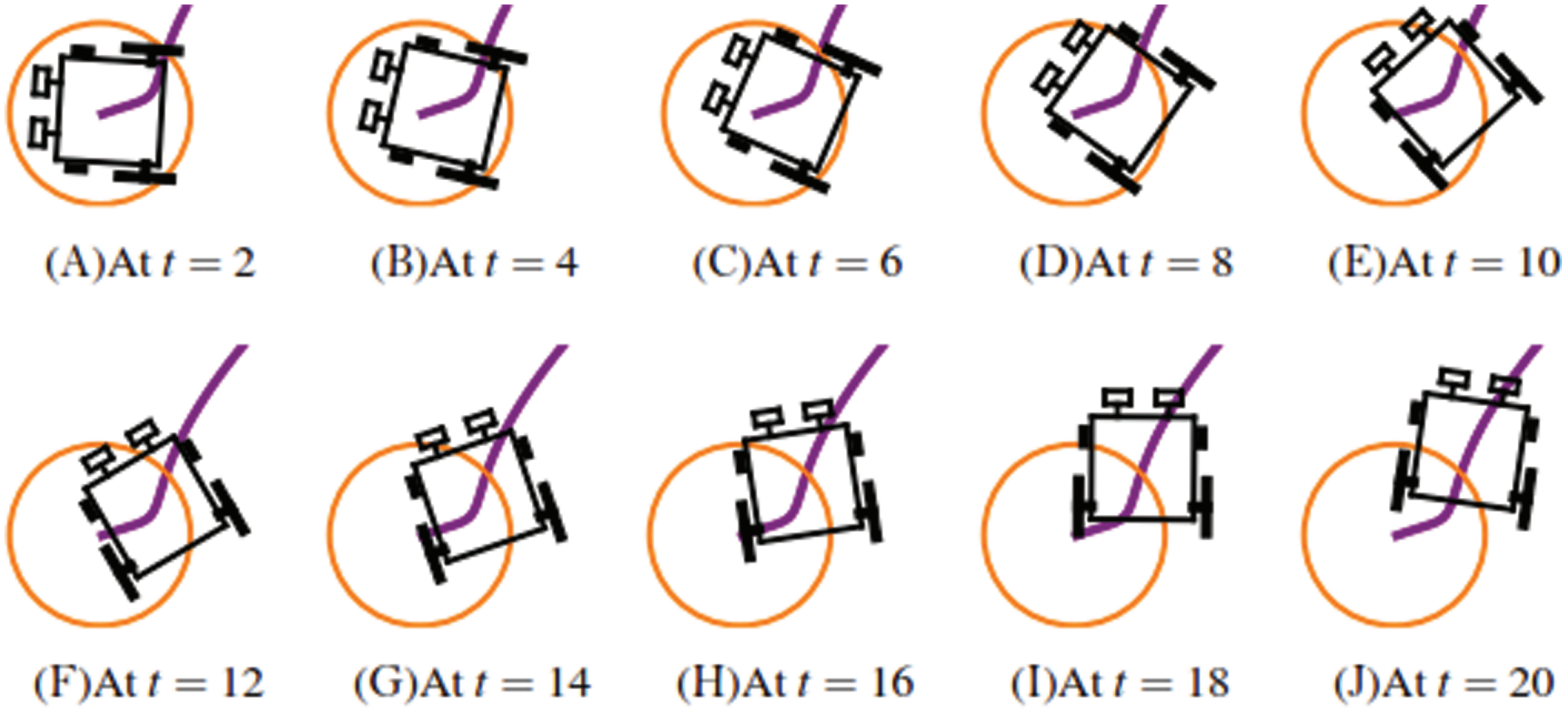
*Example 1*. Images taken at *t* = 2, 4, 6, 8, 10, 12, 14, 16, 18 and 20 respectively showing the rotational motion of the wheelchair robot.

**Example 1.** The wheelchair robot has to maneuver to its target avoiding the obstacle in its way. For this example, Table 1 shows the numerical values of the initial states, constraints, and control and convergence parameters used for the wheelchair. As time evolves the robot moves to its target as shown in Fig. 7. Figure 8 shows the evolution of the monotonically decreasing $L({\bf x})$ and its time derivative. This indicates that the wheelchair robot is converging to its target. The angular velocities, ${\upsilon _R}$ and ${\upsilon _L}$ of the wheelchair robot is shown in Fig. 9. The negative angular velocities of the wheels indicate that the wheels are turned in the reverse direction and there is rapid deceleration wheelchair robot approaches the target. The linear velocities of the right and left wheels of the wheelchair robot is shown in Fig. 10. In Fig. 11 snapshots had been taken which shows the rotational motion of the wheelchair robot.

**Table 1:** *Example 1*. Numerical values of the initial states, constraints, and control and convergence parameters of the wheelchair robot.

Initial configuration	
Rectangular position	(*x*_0_, *y*_0_) = (10, 10)
Initial orientation, *θ* rad	Randomly generated
**Constraints**	
Dimensions	*ζ* = 5, *r* = 2, *η* = 3
Target	(*a*, *b*) = (100, 100)
Fixed obstacle	(*o*_11_, *o*_12_) = (50, 50)
Radius of fixed obstacle	*r*_*o*1_ = 10
Maximum angular velocities	*υ*_*Rmax*_ = *υ*_*Lmax*_ = 1
Parameter used to bound *υ*_*R*_ and *υ*_*L*_	*χ* = 1
**Control parameters**	
Target convergence	*α* = 2
Obstacle avoidance	*β*_1_ = 0.01

**Figure 12:**
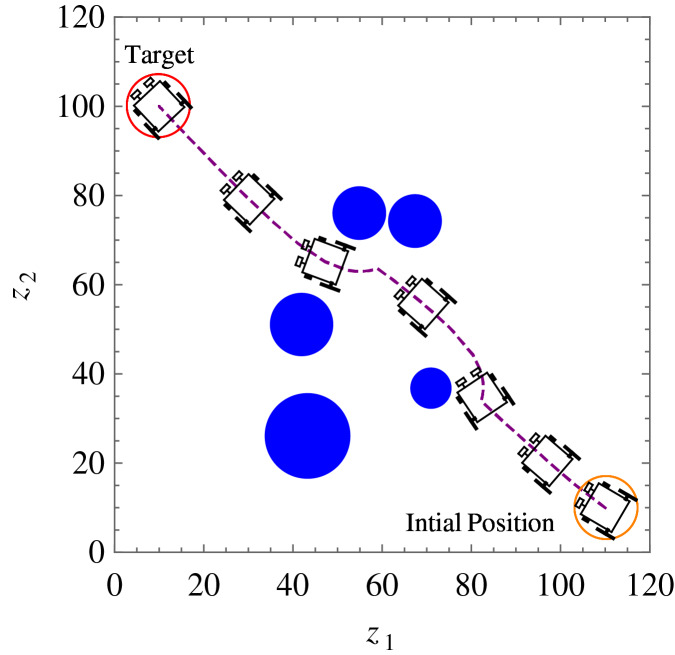
*Example 2*. Position and orientation of the wheelchair robot at *t* = 0, 2, 8, 19, 31, 34 and 50 respectively. The trajectory of (x, y) is shown in dashed line.

**Figure 13:**
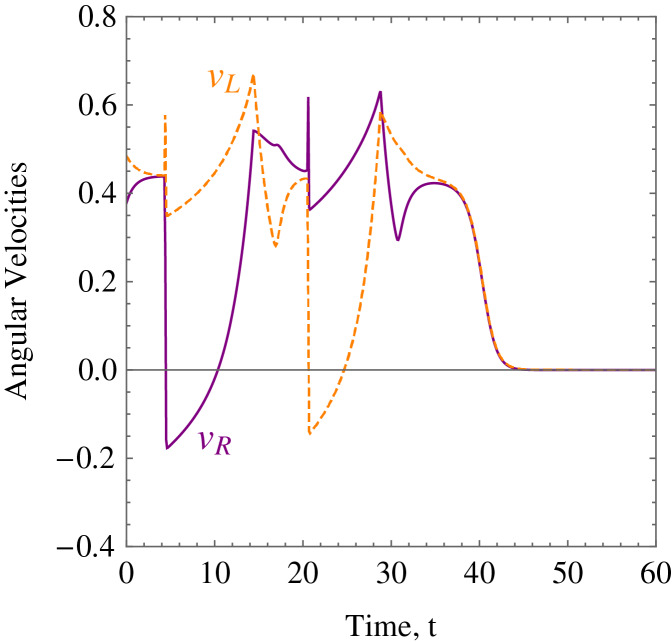
*Example 2*. The angular velocities of the wheelchair robot showing rapid deceleration as it approaches the target.

**Example 2.** Five static obstacles were randomly generated, and the wheelchair robot has to avoid those that fall on its path in its way to its target. Table 2 only shows the numerical values of the initial states, constraints, and control and convergence parameters of the wheelchair robot which were different from *Example 1* for this example. The position at different times of wheelchair robot as it maneuvers to its target avoiding the obstacles in its way is shown in Fig. 12. The angular velocities, ${\upsilon _R}$ and ${\upsilon _L}$ of the wheelchair robot is shown in Fig. 13. The negative velocities of the wheels indicate that the wheels are turned in the reverse direction and there is rapid deceleration wheelchair robot approaches the target. The evolution of $L({\bf x})$ and its time derivative are similar to that of *Example 1*. Monotonically decreasing $L({\bf x})$ indicates that the wheelchair robot is converging to its target.

**Table 2:** *Example 2*. Numerical values of the initial states, constraints, and control and convergence parameters of the wheelchair robot.

Initial configuration	
Rectangular position	(*x*_0_, *y*_0_) = (110, 10)
**Constraints**	
Target	(*a*, *b*) = (10, 100)
Fixed obstacles	Five were (*q* = 5) randomly generated
Radius of fixed obstacle	Randomized between 2 and 10
Obstacle avoidance	*β*_*k*_ = 0.001 for *k* = 1, 2, 3, …, *q*

**Figure 14:**
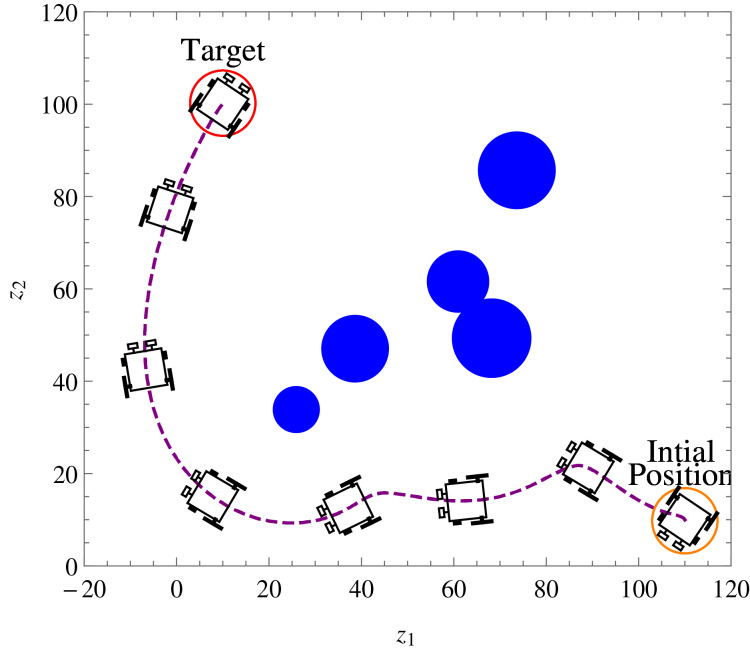
*Example 3*. Position and orientation of the wheelchair robot at *t* = 0, 45, 95, 140, 190, 230, 270 and 360 respectively. The trajectory of (x, y) is shown in dashed line.

**Figure 15:**
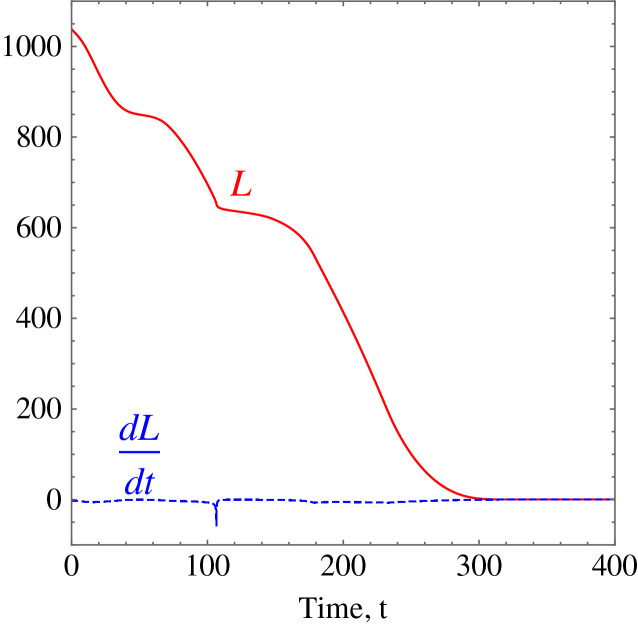
*Example 3*. Monotonically decreasing Lyapunov function and its time derivative.

**Figure 16:**
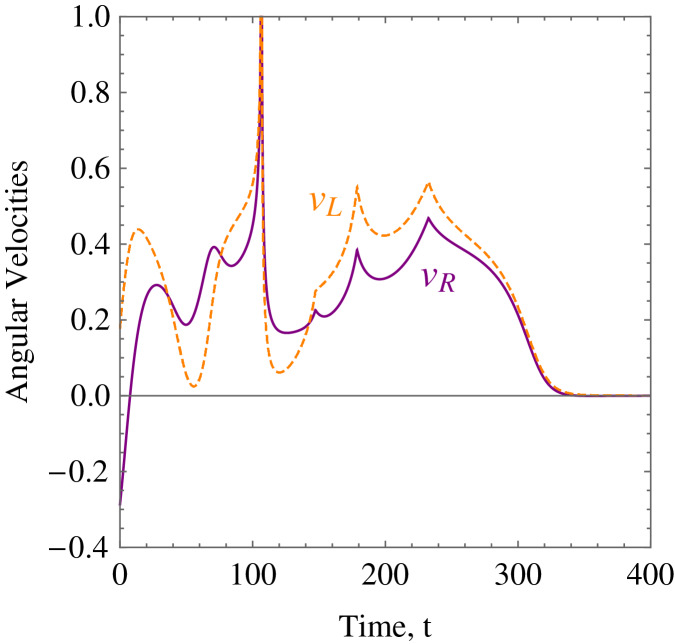
*Example 3*. The angular velocities of the wheelchair robot showing rapid deceleration as it approaches the target.

**Example 3.** Five random obstacles were generated in the workspace of the wheelchair robot. The robot has to maneuver to its target avoiding the obstacle in its way. For this example, Table 2 shows the numerical values of the initial states, and constraints. The control parameter for target convergence is 0.1 whereas the obstacle avoidance parameter was randomised between 3 and 10. As time evolves the robot moves to its target as shown in Fig. 14. Figure 15 shows the evolution of the monotonically decreasing $L({\bf x})$ and its time derivative. This indicates that the wheelchair robot is converging to its target. The angular velocities, ${\upsilon _R}$ and ${\upsilon _L}$ of the wheelchair robot is shown in Fig. 16. The negative velocities of the wheels indicate that the wheels are turned in the reverse direction and there is rapid deceleration wheelchair robot approaches the target.

## Discussion

The introduction of smart wheelchairs is making our society socially and economically inclusive for PWDs. In this paper, a set of nonlinear, time-invariant, continuous, and stabilizing velocity controllers of a wheelchair robot has been established to navigate in an obstacle-ridden environment while observing system restrictions and limitations. Simulation results such as the ones shown in Figs. 7, 12, and 14 show the controllers’ effectiveness and system robustness for navigation in an obstacle-ridden environment.

This has provided a solution to the common problem tagged to wheelchairs that require human interactions whereby persons with special disabilities are incapable of any interactions or are not able to provide any commands to the wheelchairs. For instance, for those who have severe paralysis, mental impairment, concomitant impairments, musculoskeletal problems, and spinal cord injury patients, even the simplest of the tasks requiring moving from one place to the other are being compromised. When we look at the smart wheelchair proposed in this paper, this problem is resolved where the person using the wheelchair is not required to provide any command, and navigation or the task can be controlled centrally. The system proposed suits a user’s routine activities, which could be pre-programmed as the central command. For instance, at a specific time, the user should be heading for the dining area, washroom, tea room, and so on, enabling the user to move around for scheduled activities without assistance from another person. Whereas for ad-hoc activities, a user or another person (where the user is not capable) has to interact with the wheelchair to provide the command for navigation.

In comparison to the systems presented in Borges et al. (2018), Gulpanich, Petchhan & Wongvanich (2018), Solea et al. (2015), Saitoh, Takahashi & Konishi (2007), Tomari, Kobayashi & Kuno (2014), Xin et al. (2018), Qin, Li & Nawaz (2018), Yashoda et al. (2018), and Shahnaz et al. (2017) the current system is stable; shows system robustness, and most importantly, it navigates autonomously, whereas the systems presented in Borges et al. (2018), Gulpanich, Petchhan & Wongvanich (2018), Solea et al. (2015), Saitoh, Takahashi & Konishi (2007), Tomari, Kobayashi & Kuno (2014), Xin et al. (2018), Qin, Li & Nawaz (2018), Yashoda et al. (2018), and Shahnaz et al. (2017) require user interaction. However, the major drawback of LbCS based on the classical approach of artificial potential field technique is the algorithm singularities or local minima. To add on, the sharp change in angular velocities of the wheels is a limitation of this study as well. Fine-tuning the control parameters would minimize the sharp change of velocities of the two wheels to a certain extend. However, to ensure that there is no sharp change in velocities of the wheels, there is a need to mathematically optimize the tuning parameters, which is an open field of study.

## Conclusion

Stabilizing two-dimensional velocity-based controllers were proposed for two rear wheels driven nonholonomic wheelchair with two front castor wheels. The nonlinear time-invariant continuous controllers enabled the wheelchair, governed by its kinematic equations, to navigate from its initial configuration to a target location in an obstacle-ridden environment while observing the system restrictions and limitations. Interaction of the three main pillars of LbCS, which are safety, shortness, and smoothest path for motion planning, bring about cost and time effectiveness and efficiency of the velocity controllers. From the authors’ point of view, this is the first time such stabilizing continuous velocity-based controllers are derived for autonomous wheelchairs in Lyapunov’s sense.

This paper is a theoretical exposition into the applicability of LbCS, and we have restricted ourselves to showing the effectiveness of velocity-based control laws using computer-based simulations of interesting scenarios and numerical proofs. The drawback of this approach is that algorithm singularities (local minima) can be introduced. In practical applications, continuity has to be discretized, and only asymptotic stability could be shown. It is feasible for the industry sector to include such controllers for the development of autonomous wheelchairs. The development of such assistive technologies, which are affordable, can accelerate PWD’s social and economic inclusion.

The future work will consider combining the current algorithm, however, with acceleration controllers to one of the heuristic-based approaches to form a hybrid system, which inherits the benefits of LbCS but can flush out local minima using the latter approach. The acceleration controllers will increase the comfort level of the user of the wheelchair. Furthermore, motion planning and formation control of multiple nonholonomic wheelchairs for real-like applications will also be considered.

## Supplemental Information

10.7717/peerj-cs.725/supp-1 Supplemental Information 1 Simulation Codes.

## Additional Information and Declarations

### Data Availability

The following information was supplied regarding data availability: The code is available in the Supplemental File.
